# Ayurveda in Contemporary Clinical Practice: Integrating Traditional Principles With Preventive, Metabolic, and Mind–Body Medicine

**DOI:** 10.7759/cureus.113836

**Published:** 2026-08-02

**Authors:** Rupendra Chandrakar, Subhabrata Sarkar, Archana V Panchaxarimath, Varsha Paikra, Ajai Kumar Pandey, Sushma Rajwade

**Affiliations:** 1 Department of Samhita Siddhant, Government Ayurved College and Hospital, Pt. Deendayal Upadhyay Memorial Health Sciences and Ayush University of Chhattisgarh, Raipur, IND; 2 Department of Kaumarbhritya-Balroga, Institute of Medical Sciences (IMS), Banaras Hindu University (BHU), Varanasi, IND; 3 Department of Swasthavritta and Yoga, Parul Institute of Ayurveda and Research, Parul University, Vadodara, IND; 4 Department of Kayachikitsa, Government Ayurved College and Hospital, Pt. Deendayal Upadhyay Memorial Health Sciences and Ayush University of Chhattisgarh, Raipur, IND; 5 Department of Kayachikitsa, Institute of Medical Sciences (IMS), Banaras Hindu University (BHU), Varanasi, IND

**Keywords:** ayurveda, integrative medicine, metabolic health, mind-body medicine, preventive medicine

## Abstract

The increasing burden of chronic, metabolic, and stress-related disorders has created a need for clinical models that emphasize prevention, lifestyle modification, individualized care, and long-term risk reduction. Ayurveda offers a structured framework for diet, behavior, metabolic regulation, constitutional assessment, and mind-body balance; however, its integration into contemporary clinical practice remains constrained by fragmented evidence, heterogeneous interventions, and variable methodological quality. This review examines the relevance of Ayurveda in preventive, metabolic, and mind-body medicine and evaluates how traditional principles may be aligned with evidence-based clinical care. A structured narrative review was conducted using PubMed/MEDLINE, Scopus, Web of Science, and Google Scholar to identify primarily English-language literature published between January 2015 and June 2026. Original clinical studies, randomized trials, observational and translational studies, systematic reviews, methodological studies, and relevant policy documents were selected according to their relevance to preventive, metabolic, mind-body, personalized, safety, and regulatory aspects of Ayurveda. The review was intended as an illustrative narrative synthesis rather than a comprehensive systematic review. The evidence suggests that Ayurveda is most appropriately positioned as an adjunctive framework for cardiometabolic risk reduction, diabetes and metabolic syndrome management, mood and sleep regulation, chronic pain care, and personalized prevention. Emerging fields such as Ayurgenomics, Ayurinformatics, microbiome research, and systems biology may support the translation of traditional constructs such as *Prakriti* into testable biomedical models. Responsible integration requires rigorous trial design, standardized formulations, transparent reporting, pharmacovigilance, and regulatory oversight. Overall, Ayurveda may contribute to patient-centered preventive and integrative care when applied through evidence-informed, safety-conscious, and clinically accountable models.

## Introduction and background

Chronic, metabolic, musculoskeletal, and stress-related disorders contribute substantially to the global burden of disability and increasingly affect populations in low- and middle-income countries, creating a need for long-term preventive and therapeutic strategies. The Global Burden of Disease Study 2021 identified noncommunicable diseases, mental health conditions, musculoskeletal disorders, and metabolic diseases as major contributors to years lived with disability and disability-adjusted life-years [1]. Important modifiable contributors include unhealthy dietary patterns, elevated body mass index, hyperglycemia, hypertension, tobacco exposure, environmental risks, and low physical activity [2]. These trends support clinical models that extend beyond episodic treatment toward prevention, early risk modification, resilience, and long-term patient-centered care.

This context has renewed interest in traditional, complementary, and integrative medicine. The World Health Organization (WHO) has emphasized that traditional systems should be evaluated through appropriate assessment of safety, effectiveness, quality assurance, and evidence-based implementation [3]. The WHO Global Centre for Traditional Medicine represents a policy-level initiative intended to support the systematic study of traditional knowledge systems and the identification of validated practices that may be responsibly incorporated into healthcare systems [4]. Within this broader context, Ayurveda remains relevant because it offers a framework for health maintenance, individualized care, dietary and lifestyle regulation, and mind-body balance.

Contemporary scientific interest in Ayurveda is increasingly linked to the critical evaluation of Ayurvedic formulations and practices through modern pharmacology, toxicology, analytical science, and clinical research methods. Ayurvedic herbal formulations may contain bioactive compounds with therapeutic potential, but their clinical application requires careful evaluation of safety, efficacy, standardization, quality control, and mechanisms of action [5]. The transition from traditional textual knowledge to reproducible clinical application has been described as challenging but promising, particularly in relation to phytochemical characterization, biological validation, and clinically meaningful outcomes [6]. Historical use alone, therefore, is not sufficient to justify clinical integration without appropriate scientific evaluation.

The COVID-19 period further increased discussion around Ayurveda and other AYUSH systems in relation to public health preparedness, prevention, and integrative clinical service delivery. Ministry of AYUSH initiatives during this period contributed to research mobilization and public-health-oriented interest in traditional interventions [7]. Broader integrative approaches focused on diet, physical activity, sleep, stress management, and behavioral risk reduction are also relevant to public health practice and Sustainable Development Goal 3 [8]. Ayurveda has also been used outside India in settings related to health promotion, self-care, lifestyle modification, and perceived well-being, as illustrated by a mixed-methods case study from Sweden; such evidence is best interpreted as contextual rather than definitive clinical evidence [9]. In India, institutional recognition of Ayurveda and yoga is reflected in their inclusion within national programs addressing cancer, diabetes, cardiovascular disease, and stroke [10].

A central concept in Ayurveda is Prakriti, which refers to an individual’s constitutional profile. This is traditionally described through varying expressions of Vata, Pitta, and Kapha, often referred to as Doshas, which are theoretical functional constructs within Ayurveda rather than established biomedical entities. Another important Ayurvedic concept is Agni, which broadly refers to digestive and metabolic functioning. Emerging research has explored whether Prakriti classifications correspond to measurable biological variation, including gut and oral microbiome patterns [11]. Prakriti has also been discussed as a possible framework relevant to predictive, preventive, and personalized medicine, although reproducible assessment tools, biological validation, and outcome-linked clinical evidence remain necessary [12]. Related translational fields such as Ayurgenomics seek to examine possible links between Ayurvedic constitutional classifications and genomic, molecular, metabolic, sensory, or microbiome-related variation.

The contemporary evidence base for Ayurveda remains heterogeneous. Registered Ayurveda trials have highlighted the need for customized reporting elements that adequately capture Ayurvedic diagnostic categories, individualized treatment logic, formulations, procedures, and outcomes [13]. Early clinical evidence includes an AYUSH integration project evaluating Ayurveda intervention, lifestyle modification, and yoga in prediabetes and type 2 diabetes [14]. Recent policy analysis of the WHO Global Traditional Medicine Strategy 2025-2034 has further emphasized strengthening research evidence, regulatory mechanisms, safety and quality assurance, and the responsible integration of validated traditional, complementary, and integrative medicine into national health systems [15]. These developments indicate growing clinical and policy interest but do not remove the need for cautious interpretation, particularly because the evidence base includes randomized trials, observational studies, protocols, translational research, policy analyses, and case-level evidence of varying methodological strength.

**Figure 1:**
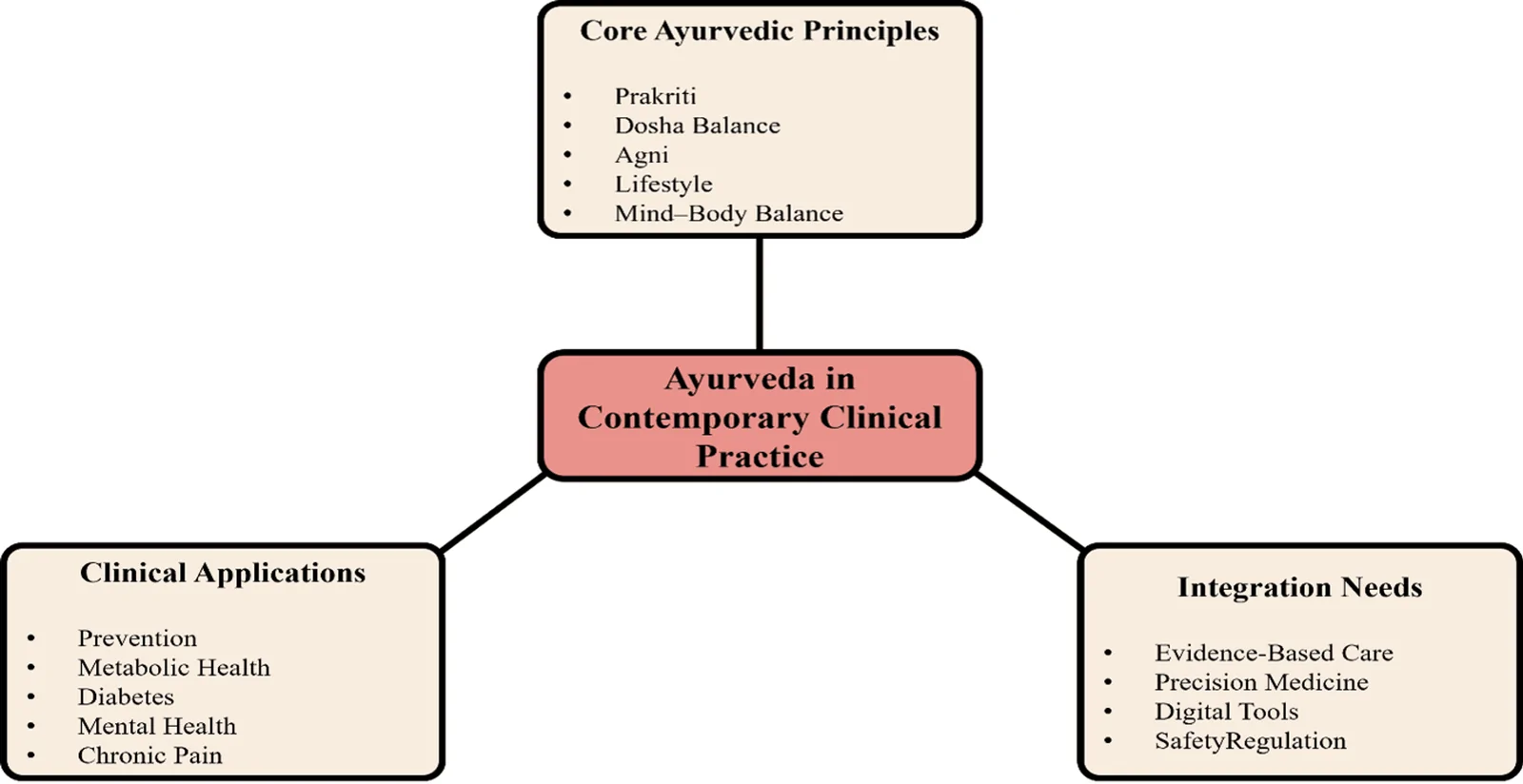
Framework for integrating Ayurveda into contemporary clinical practice. Created by the authors using Microsoft PowerPoint.

In this review, contemporary clinical practice refers to the structured, monitored, and safety-conscious application of Ayurveda within preventive care, cardiometabolic management, diabetes and metabolic syndrome care, mind-body regulation, personalized assessment, and broader integrative healthcare. Ayurveda-specific evidence includes studies evaluating Ayurvedic formulations, Prakriti-based assessment, whole-system Ayurvedic protocols, or Ayurvedic procedures. Yoga-only and general lifestyle interventions are not treated as direct evidence of Ayurveda; when included, they are presented as complementary contextual evidence within the broader integrative-care framework. Figure 1 summarizes the proposed framework for integrating Ayurveda into contemporary clinical practice.

Objectives of the review

This review aims to examine the role of Ayurveda in preventive, metabolic, and mind-body medicine; compare selected traditional principles with contemporary approaches to lifestyle medicine, individualized care, and chronic disease prevention; summarize emerging clinical and translational evidence; and identify priorities related to safety, standardization, regulation, and future research.

## Review

Methodology

This narrative review was prepared using a focused and structured literature search. PubMed/MEDLINE, Scopus, Web of Science, and Google Scholar were consulted for relevant English-language publications, primarily from January 2015 to June 2026. Selected earlier publications and policy documents were included when they provided important background, regulatory guidance, or conceptual context. The principal search terms included “Ayurveda,” “preventive medicine,” “metabolic health,” “diabetes,” “metabolic syndrome,” “mind-body medicine,” “Prakriti,” “personalised medicine,” “Ayurgenomics,” “safety,” “standardisation,” and “regulation,” used individually and in combination. Original clinical studies, randomized controlled trials, observational and translational studies, systematic reviews, methodological studies, and relevant policy documents were considered. Publications were selected according to their relevance to the review objectives, with preference given to human studies addressing preventive, metabolic, psychological, personalized, safety, and regulatory applications of Ayurveda. Articles unrelated to the review scope, duplicate reports, conference abstracts with insufficient information, and purely historical or philosophical discussions without clear contemporary clinical relevance were excluded. The selected literature was organized into thematic sections. This review was intended as a structured narrative synthesis rather than a comprehensive systematic review; therefore, formal Preferred Reporting Items for Systematic Reviews and Meta-Analyses (PRISMA) reporting and risk-of-bias scoring were not undertaken. No meta-analysis, meta-regression, or pooled statistical synthesis was performed because the included studies differed substantially in populations, interventions, comparators, study designs, treatment durations, and reported outcomes. Findings were synthesized narratively, with study designs and evidence types identified and conclusions interpreted cautiously according to their methodological strength.

Foundational principles of Ayurveda and their relevance to modern medicine

Prakriti is a central Ayurvedic concept describing an individual’s constitutional profile and is traditionally used to guide personalized dietary, behavioral, and therapeutic recommendations. Contemporary translational research has examined whether Prakriti classifications correspond to measurable biological variation. Shalin et al. reported differences in gut and oral microbiome signatures among individuals with specific Prakriti types [11]. Jnana et al. examined Prakriti phenotypes as potential stratifiers of gut microbiome variation [16]. Saini et al. investigated Prakriti-based differences in olfactory perception and receptor gene expression [17]. Trivedi et al. applied an Ayurgenomic approach to Duchenne/Becker muscular dystrophy and explored its possible relevance to individualized supportive care [18]. These findings suggest that Prakriti may serve as a hypothesis-generating framework for translational research; however, standardized assessment tools, reproducible biological validation, and evidence of improved clinical outcomes are required before routine application.

Ayurveda in preventive and metabolic medicine

Preventive and Lifestyle-Based Care

The preventive relevance of Ayurveda has been explored in studies involving prediabetes, type 2 diabetes, obesity, and metabolic syndrome. Sharma et al. evaluated an integrated program combining Ayurveda, lifestyle modification, and yoga in individuals with prediabetes and type 2 diabetes under the National Programme for Prevention and Control of Cancer, Diabetes, Cardiovascular Diseases and Stroke-AYUSH integration project, suggesting the feasibility of a combined prevention-oriented model within chronic disease programs [14]. Kumari et al. assessed an integrated Ayurveda treatment protocol in uncontrolled type 2 diabetes mellitus through a randomized controlled study and reported possible clinical relevance for a multidimensional intervention model [19]. Padhar et al. evaluated Arogyavardhini compound with lifestyle modification in metabolic syndrome through a double-blind, placebo-controlled randomized clinical trial, suggesting potential adjunctive relevance alongside structured lifestyle correction [20]. Munshi et al. assessed Nisha-Amalaki capsules in prediabetic patients through a randomized controlled proof-of-concept study and reported findings relevant to glycemic risk reduction and short-term safety [21]. Collectively, these studies suggest that Ayurveda may have potential value as a monitored adjunct within prevention-oriented metabolic care rather than as an isolated or unsupervised intervention. Table 1 summarizes selected evidence related to preventive and lifestyle-based Ayurvedic care.

**Table 1:** Preventive medicine applications of Ayurveda in contemporary clinical care.

Theme	Clinical relevance	Evidence/Interpretation	References
Integrated Ayurveda care in type 2 diabetes	Supports prevention-oriented management of cardiometabolic risk through diet, lifestyle, metabolic correction, and individualized therapy	A structured Ayurveda protocol showed possible clinical relevance in uncontrolled type 2 diabetes, suggesting greater relevance as a multidimensional care model rather than an isolated herbal treatment	[19]
Ayurvedic biology and Prakriti-based translational evidence	Provides a scientific bridge between classical Ayurvedic concepts and modern preventive medicine	Original microbiome and Ayurgenomics studies suggest that Prakriti-based phenotypes may be linked with measurable microbial, sensory, and molecular variation	[11,18]
Ayurveda and metabolic syndrome	Relevant to early risk modification in obesity, insulin resistance, dyslipidemia, and cardiometabolic clustering	A placebo-controlled randomized trial of Arogyavardhini compound with lifestyle modification supports Ayurveda as an adjunct to structured lifestyle intervention	[20]
Clinical trial infrastructure	Important for future validation of Ayurveda-based preventive interventions	Ayurveda trial registration analysis highlights the need for transparent registration, standardized endpoints, disease-specific protocols, customized data elements, and stronger methodological consistency	[13]

Ayurvedic Nutrition and Metabolic Health

Clinical studies have explored Ayurveda-based interventions in metabolic syndrome, obesity, prediabetes, type 2 diabetes, and nonalcoholic fatty liver disease. Chandake et al. conducted a randomized double-blind controlled trial of Tryushnadya Churna in metabolic syndrome with obesity and reported improvements in selected metabolic parameters [22]. Munshi et al. evaluated Nisha-Amalaki capsules in prediabetes using a randomized controlled proof-of-concept design, suggesting potential efficacy and acceptable short-term safety that require confirmation in larger studies [21]. Kumari et al. assessed an integrated Ayurveda protocol for uncontrolled type 2 diabetes in a randomized controlled study, suggesting possible adjunctive relevance for a multimodal approach involving diet, lifestyle regulation, and metabolic correction [19]. A recent critical review identified substantial variation in Prakriti assessment methods and found that most available tools lacked adequate validation, reliability testing, and evaluation across diverse populations, limiting their current clinical application [23]. Prakriti should therefore be regarded as a research-oriented phenotypic framework rather than an established clinical biomarker. Remya et al. evaluated Sharapunkhadi powder with lifestyle modification in nonalcoholic fatty liver disease through a randomized placebo-controlled trial, supporting further evaluation of this combined approach [24]. Interpretation remains limited by the relatively small and heterogeneous evidence base, the use of multimodal interventions, and the absence of a formal methodological quality assessment in this narrative review. Collectively, these findings suggest potential adjunctive relevance for Ayurveda-based nutrition and formulation-guided care within monitored metabolic management, but they do not establish equivalence or superiority to standard diagnostic monitoring, pharmacotherapy, or evidence-based metabolic care.

Ayurveda in Diabetes and Metabolic Syndrome

Diabetes and metabolic syndrome have been examined through Ayurveda-specific interventions and complementary yoga-based evidence. Krishniya et al. evaluated Trikatu Churna with lifestyle modification in Sthaulya through a placebo-controlled randomized clinical study, suggesting potential relevance for further evaluation of Ayurveda-based obesity management [25]. Sivapuram et al. examined Ayurveda body-mind constitutional types alongside a yoga intervention among individuals with type 2 diabetes mellitus, suggesting that constitution-based assessment may help individualize lifestyle and mind-body interventions [26]. Yadav et al. compared a 12-week yoga-based lifestyle program, including dietary modification, with dietary intervention alone in Indian adults with metabolic syndrome and reported findings relevant to cardiometabolic risk modification [27]. This intervention was yoga-based rather than explicitly Ayurvedic and is therefore included as complementary contextual evidence within the broader integrative-care framework. Perera et al. assessed the efficacy and safety of an Ayurveda herbal formulation in uncomplicated type 2 diabetes mellitus, suggesting potential monitored adjunctive use in glycemic care [28]. A systematic review and meta-analysis of 199 randomized trials involving 21,191 participants reported potential glycemic benefits for several Ayurvedic medicines; however, poor methodological reporting, limited certainty of evidence, and inadequate adverse event documentation prevented definitive conclusions [29]. Collectively, the Ayurveda-specific studies suggest potential adjunctive relevance for constitution-based counselling, lifestyle regulation, weight management, and selected formulations under clinical supervision, while the yoga-based study provides separate complementary evidence for cardiometabolic lifestyle management. Table 2 summarizes both Ayurveda-specific evidence and clearly labelled complementary yoga-based evidence related to diabetes and metabolic syndrome.

**Table 2:** Ayurveda-based interventions for diabetes and metabolic syndrome.

Focus area	Clinical implication	Complementary interpretation	References
Obesity and early metabolic risk	Trikatu Churna with lifestyle modification may support obesity-related risk reduction in Sthaulya	Relevant because obesity is an upstream driver of insulin resistance, dyslipidemia, hypertension, and type 2 diabetes progression	[25]
Constitution-based metabolic care	Ayurvedic body–mind constitutional assessment may help individualize lifestyle and yoga-based interventions in type 2 diabetes	Supports the Ayurvedic view that metabolic care should consider patient-specific physiology, behaviour, and mind–body profile	[26]
Yoga-based lifestyle intervention	A 12-week yoga-based programme incorporating dietary modification showed potential relevance for cardiometabolic risk reduction in adults with metabolic syndrome	This study provides complementary yoga and lifestyle evidence and should not be interpreted as direct evidence of an Ayurveda-specific intervention	[27]
Herbal therapy in type 2 diabetes	An Ayurvedic herbal formulation reported potential glycemic benefit and no major short-term safety concerns in uncomplicated type 2 diabetes	Suggests possible adjunctive glycemic benefit, requiring use within monitored clinical care rather than unsupervised substitution	[28]

Mind-body medicine in Ayurveda

Original randomized studies have explored the potential relevance of Ayurveda-specific and complementary mind-body interventions in depression, insomnia, and stress regulation. Punia et al. evaluated a whole-system Ayurveda management protocol in major depressive disorder through a randomized controlled clinical trial and reported clinical benefit when Ayurveda was delivered as an integrated care model [30]. Sarhyal et al. studied Brahmi Vati and Aswagandharista in major depressive disorder through a randomized controlled trial, suggesting potential adjunctive usefulness of selected classical formulations for depressive symptoms [31]. Verma et al. compared yoga and Ayurveda practice in insomnia using a randomized controlled trial design and reported favorable sleep-related outcomes [32]. Yadav et al. assessed yoga-based breathing practices in hospitalized COVID-19-positive patients and reported reductions in depression, anxiety, stress, and fear scores [33]. This was a yoga-based rather than Ayurveda-specific intervention and is included as complementary contextual evidence. Collectively, the Ayurveda-specific trials suggest potential adjunctive relevance for mood and sleep management, while the yoga-based study provides separate evidence concerning structured stress-regulation practices.

Personalized and precision medicine in Ayurveda

**Figure 2:**
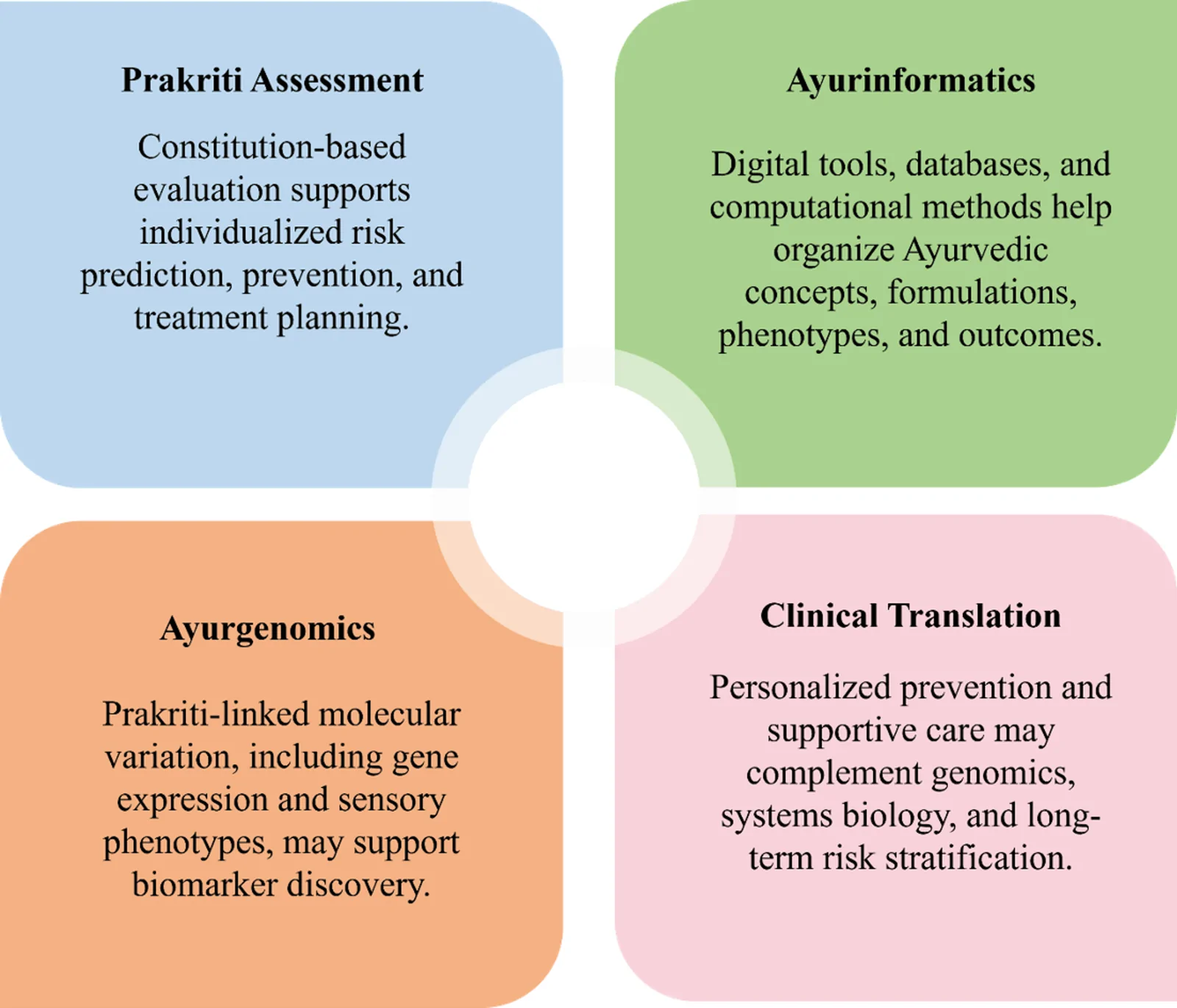
Framework for Ayurveda-based personalized care. Created by the authors using Microsoft PowerPoint.

The microbiome, sensory, molecular, and Ayurgenomic findings described above suggest that Prakriti may have potential value as a research-oriented phenotypic framework [11,16-18]. A recent critical review identified substantial variation in Prakriti assessment methods and limited validation, reliability testing, and reproducibility across populations [23]. Therefore, Prakriti should not yet be considered an established clinical biomarker or routine precision medicine tool. Figure 2 illustrates the proposed framework for Ayurveda-based personalized care.

Clinical integration of Ayurveda within contemporary healthcare systems

Clinical integration requires condition-specific protocols, transparent intervention descriptions, appropriate comparators, clinically meaningful endpoints, and adverse event monitoring. Bhatta et al. evaluated yoga with add-on Ayurvedic Kati Basti therapy for chronic low back pain through a randomized controlled trial, supporting the role of Ayurveda-linked interventions in multidisciplinary rehabilitation and non-pharmacological pain care [34]. Mata et al. presented a randomized controlled trial protocol for Ayurveda management of allergic rhinitis, indicating movement toward structured evaluation in outpatient conditions requiring long-term symptom control [35]. Yadav et al. described a double-dummy, double-blind, placebo-controlled randomized trial protocol comparing Sarpgandha Mishran with amlodipine in stage-I primary hypertension, showing that Ayurvedic interventions can be evaluated against conventional comparators using rigorous trial methods [36]. These studies illustrate possible models for structured and monitored integration; however, the allergic rhinitis and hypertension publications are trial protocols and cannot establish clinical effectiveness or safety until completed results are available. Table 3 presents selected Ayurveda-specific and directly integrative clinical models relevant to contemporary healthcare settings.

**Table 3:** Clinical integration models for Ayurveda in contemporary healthcare.

Clinical area	Integration model	Clinical relevance	Critical interpretation	References
Chronic low back pain	Yoga with add-on Ayurvedic Kati Basti	Suggests potential integration into rehabilitation, pain management, and non-pharmacological care pathways	Most relevant as adjunctive care, with functional outcomes, pain scores, quality of life, and safety requiring continued evaluation	[34]
Allergic rhinitis	Ayurveda intervention assessed through an randomized controlled trial protocol	Relevant to outpatient and primary care settings where chronic symptomatic conditions require long-term management	Protocol-level evidence demonstrates methodological feasibility but does not establish clinical effectiveness until the completed trial results are available	[35]
Stage-I hypertension	Sarpgandha Mishran compared with amlodipine using a double-dummy, double-blind randomized controlled trial design	Relevant to cardiometabolic and primary care integration	The protocol provides a rigorous Ayurveda-specific comparative design, but clinical adoption depends on completed efficacy, safety, and comparative outcome data	[36]

Safety, evidence quality, and regulatory considerations

Safety and quality control require formulation-specific evaluation rather than reliance on traditional use alone. Mohanan and Puthiyedath evaluated toxic heavy metal content in marketed Ayurvedic decoctions using closed-vessel microwave digestion and inductively coupled plasma mass spectrometry, supporting the need for contaminant testing and surveillance of marketed preparations [5]. Boini et al. developed a high-performance thin-layer chromatography-based method for simultaneous quantification of five bioactive markers in Jwarahara Kwatha Choornam, demonstrating how analytical standardization can improve reproducibility and quality assurance for polyherbal formulations [6]. Adhikari et al. analyzed Ayurveda trials registered with the Clinical Trial Registry-India and highlighted the need for customized dataset items to capture Ayurveda-specific diagnostic categories, interventions, formulations, and procedures [13]. Chaudhari and Somvanshi examined Clinical Trial Registry-India-registered Ayurveda trials for COVID-2019 and identified methodological concerns related to sample size planning, comparator selection, intervention description, outcome measures, and safety monitoring [37]. These findings show that wider clinical integration requires standardized formulations, batch-level quality testing, transparent trial registration, pre-specified endpoints, adverse event reporting, herb-drug interaction assessment, and pharmacovigilance.

Limitations and future directions

This review has a few limitations. Interpretation is constrained by small study samples, heterogeneous multimodal interventions, inconsistent comparators and outcome measures, incomplete safety reporting, and the inclusion of protocols and case-level evidence that cannot establish clinical effectiveness. The high degree of heterogeneity across Ayurveda studies, including variation in formulations, treatment duration, dosage, practitioner experience, diagnostic criteria, comparator groups, and outcome measures, further restricts the strength and generalizability of the conclusions. Several interventions are delivered as multimodal care packages involving herbal medicines, diet, lifestyle modification, yoga, counselling, and procedural therapies, making it difficult to determine the independent effect of each component. The evidence base also includes small randomized trials, methodological and protocol studies, observational studies, and condition-specific reports of varying quality. Regional differences in Ayurveda practice, product standardization, patient characteristics, and healthcare settings further limit generalizability. Safety interpretation remains constrained by incomplete reporting of adverse events, herb-drug interactions, contaminant testing, and long-term follow-up.

Future trials should focus on large, multicenter, well-designed randomized controlled trials with clear methodology, uniform formulations, and well-defined clinically relevant endpoints, thorough comparator groups, and comprehensive safety surveillance. The studies should explicitly specify the Ayurvedic diagnostic categories, the individual treatment logic, product quality control, compliance, and co-interventions. The mechanisms related to metabolism, inflammation, microbiome diversity, stress physiology, and genomics should be determined through translational research. Enhanced reproducibility and synthesis of evidence may be achieved through digital registries, Ayurinformatics platforms, and harmonized reporting standards. Clinical integration must be performed in well-defined areas of care ancillary to preventive, metabolic, and mind-body care, with the aid of pharmacovigilance, regulation, cost-benefit analysis, and implementation research.

## Conclusions

This review indicates that Ayurveda may have potential adjunctive relevance in preventive care, cardiometabolic risk management, diabetes and metabolic syndrome, mind-body regulation, chronic pain, and personalized assessment. Its contemporary clinical application is most appropriately considered within structured care models combining individual assessment, dietary and lifestyle guidance, stress and sleep management, selected formulations, and appropriate clinical monitoring. Ayurveda should not replace evidence-based conventional treatment but may complement it in carefully selected settings under qualified supervision. The available evidence remains limited by small samples, heterogeneous multimodal interventions, inconsistent outcome reporting, and incomplete long-term safety data. Emerging fields such as Ayurgenomics, Ayurinformatics, microbiome research, and systems biology may help translate selected traditional concepts into testable research frameworks, but routine clinical application requires further validation. Responsible integration will depend on rigorous comparative trials, standardized formulations, transparent reporting, pharmacovigilance, regulatory oversight, and implementation research.
